# Homodimerization of Amyloid Precursor Protein at the Plasma Membrane: A homoFRET Study by Time-Resolved Fluorescence Anisotropy Imaging

**DOI:** 10.1371/journal.pone.0044434

**Published:** 2012-09-04

**Authors:** Viviane Devauges, Catherine Marquer, Sandrine Lécart, Jack-Christophe Cossec, Marie-Claude Potier, Emmanuel Fort, Klaus Suhling, Sandrine Lévêque-Fort

**Affiliations:** 1 Institut des Sciences Moléculaires d’Orsay, CNRS UMR 8214, Univ. Paris Sud, Orsay, France; 2 Laboratoire Charles Fabry, CNRS UMR 8501, Institut d’Optique, Univ. Paris Sud, Palaiseau, France; 3 Centre de Photonique Biomédicale, Univ. Paris Sud, CLUPS/LUMAT FR2764, Orsay, France; 4 CRICM UPMC/INSERM UMR-S975/CNRS UMR 7225, Paris, France; 5 Institut Langevin, ESPCI ParisTech, Univ. Paris Diderot, UMR 7587, Paris, France; 6 Department of Physics, King’s College London, Strand, London, United Kingdom; University of Melbourne, Australia

## Abstract

Classical FRET (Förster Resonance Energy Transfer) using two fluorescent labels (one for the donor and another one for the acceptor) is not efficient for studying the homodimerization of a protein as only half of the homodimers formed can be identified by this technique. We thus resorted to homoFRET detected by time-resolved Fluorescence Anisotropy IMaging (tr-FAIM). To specifically image the plasma membrane of living cells, an original combination of tr-FAIM and Total Internal Reflection Fluorescence Lifetime Imaging Microscope (TIRFLIM) was implemented. The correcting factor accounting for the depolarization due to the high numerical aperture (NA) objective, mandatory for TIRF microscopy, was quantified on fluorescein solutions and on HEK293 cells expressing enhanced Green Fluorescence Protein (eGFP). Homodimerization of Amyloid Precursor Protein (APP), a key mechanism in the etiology of Alzheimer’s disease, was measured on this original set-up. We showed, both in epifluorescence and under TIRF excitation, different energy transfer rates associated with the homodimerization of wild type APP-eGFP or of a mutated APP-eGFP, which forms constitutive dimers. This original set-up thus offers promising prospects for future studies of protein homodimerization in living cells in control and pathological conditions.

## Introduction

Protein interactions are central to most of physiological and pathological processes and studying them dynamically is one of the challenges of current biology. FRET imaging is an sensitive and widely used technique to monitor proteins interactions in living cells [1]. The energy of the donor can be transferred to the acceptor if they are in close proximity [2]. Three conditions must be fulfilled in order for FRET to occur: (i) there must be a spectral overlap between the emission spectrum of the donor and the absorption spectrum of the acceptor, (ii) the dipoles of emission of the donor and of absorption of the acceptor must not be perpendicular and (iii) the donor and acceptor must be within 10 nm of each other. FRET can be measured *via* several techniques. The most straightforward is to follow the increase of fluorescence intensity of the acceptor. This is, however, difficult to quantify and prone to multiple artifacts. FRET can also be measured by Fluorescence Lifetime Imaging Microscopy (FLIM). A decrease of fluorescence lifetime of the donor will be observed in case of a non-radiative energy transfer [3]–[5]. As FLIM is independent of fluorophore concentration and can be used to monitor small changes in fluorescence lifetime, it is currently considered the most accurate technique to probe protein hetero-dimerization [6]. However, this technique of classical FRET using two fluorescent labels (one for the donor and another one for the acceptor) is less efficient for studying the homodimerization or multimerization of a protein. In fact, only half of the homodimers formed can be identified by this technique.

This drawback can be overcome with fluorescence anisotropy which can be used to follow the whole set of homodimers all labeled with the same fluorescent dye, which is also easier from the genetic engineering point of view. In such conditions, homodimerization will lead to a non radiative energy transfer between the donor and the acceptor. The resulting fluorescence emission will be depolarized and consequently it will lead to a reduction of the fluorescence anisotropy [7], [8]. Biological applications include studying lipid rafts by detecting and sizing GPI-anchored proteins clusters, following protein homodimerization in the presence or absence of specific ligands or conformational changes [9]–[14].

Here we applied tr-FAIM to the homodimerization of Amyloid Precursor Protein (APP), a key player in Alzheimer’s disease (AD). AD is the most common form of dementia worldwide [15]. One of its neuropathological hallmarks is the presence of senile plaques in the brain of AD patients [16]. The main component of senile plaques is amyloid peptide (A*β*). A*β* is derived from the successive cleavage of the transmembrane protein APP (Amyloid Precursor Protein) by two transmembrane enzymes, the *β*-secretase and the *γ*-secretase complex [17]. Though central to AD, the physiological roles of APP remain elusive. As most type I transmembrane proteins, APP has an ability to dimerize [18]. However the role of APP dimerization in APP processing and A*β* production remains controversial. Indeed, most studies showed that APP dimerization increased A*β* production [19], [20] but it was also demonstrated that APP dimerization could lead to decreased A*β* production [21]. Clarifying this issue is crucial prior to developing new therapeutic strategies based on APP dimerization.

In this article, we present the implementation of time-resolved Fluorescence Anisotropy IMaging (tr-FAIM) on a TIRFLIM set-up to study protein homodimerization at the plasma membrane of living cells. We first validated our new set-up using fluorescein solutions of different viscosities. To get a dynamic follow-up of protein interactions from the plasma membrane to the cytoplasm, samples were excited both in epifluorescence and in TIRF mode, which requires high numerical aperture (NA) objectives. These objectives induce a supplementary depolarization factor in the detection pathway [22]. We thus experimentally quantified the correcting factor associated with these objectives and assessed its impact on homoFRET measurements in human HEK293 cell lines expressing wild type enhanced Green Fluorescent Protein (eGFP) or an eGFP-eGFP tandem. Taking into account this correction, we then applied tr-FAIM to the homodimerization of APP.

## Materials and Methods

### Plasmids and Reagents

pEGFP-N1 plasmid (Clontech, Mountain View, CA) was used to express eGFP in the cytoplasm. Plasmid expressing eGFP-eGFP tandem was a kind gift from J-C Mevel (Institut Jacques Monod, Université Paris 7, France). APP_751_ plasmid was a gift from Dr Frederic Checler (IPMC, Valbonne, France). The human APP_751_ sequence was inserted in the pEGFP-N1 plasmid in the AgeI/XmaI site to generate the APP-eGFP plasmid. APP-L17C-eGFP was obtained using the Quickchange mutagenesis kit (Stratagene, Santa Clara, CA). Mutation APP-L17C was previously described in [19].

Fluorescein (Fluorescein 548, MW 401.20) was purchased from Exciton (Dayton, OH) and Glycerol from Fisher Scientific (Waltham, MA). Fluorescein solutions of varying viscosity were used. To accurately determine the percentage of glycerol contained in these solutions and deduce their viscosity, we first measured the optical refractive index of the solutions with a refractometer (Atago, Japan) and then deduced the percentage of glycerol in the solutions and their viscosities using tables linking these parameters [23]. Measurements were performed at 20±2°C.

### Cell Lines

HEK293 (Human Embryonic Kidney from ATCC (Manassas, VA; CRL-1573)) cells were grown in DMEM medium supplemented with 10% fetal bovine serum and 1% penicillin G (100 U/ml)/streptomycin (100 µg/ml) +1% L-glutamine. Cells were transfected with plasmids using a DNA/Effectene mixture (Qiagen, Hilden, Germany) and were maintained at 37°C in a humidified 5% CO_2_ atmosphere.

### Time-resolved Anisotropy Imaging Microscope (tr-FAIM)

Time-resolved anisotropy measurements were performed on a Total Internal Reflection Fluorescence Lifetime Microscope described in details elsewhere [24]. Briefly, Total Internal Reflection Fluorescence Microscopy was combined with Fluorescence Lifetime Imaging to observe biological interactions occurring at or just below the plasma membrane, with a sub-wavelength axial resolution in wide-field. At the interface between the sample and the coverslip, for an angle larger than the critical angle, one can excite an evanescent wave that decreases exponentially with a characteristic length which depends on the illuminating wavelength, the refractive indeces of the media and the angle of illumination. The penetration depth allows subwavelength axial resolution. Only fluorophores located very close to the glass coverslip are excited, i.e. mainly those in the cell’s plasma membrane.

**Figure 1:**
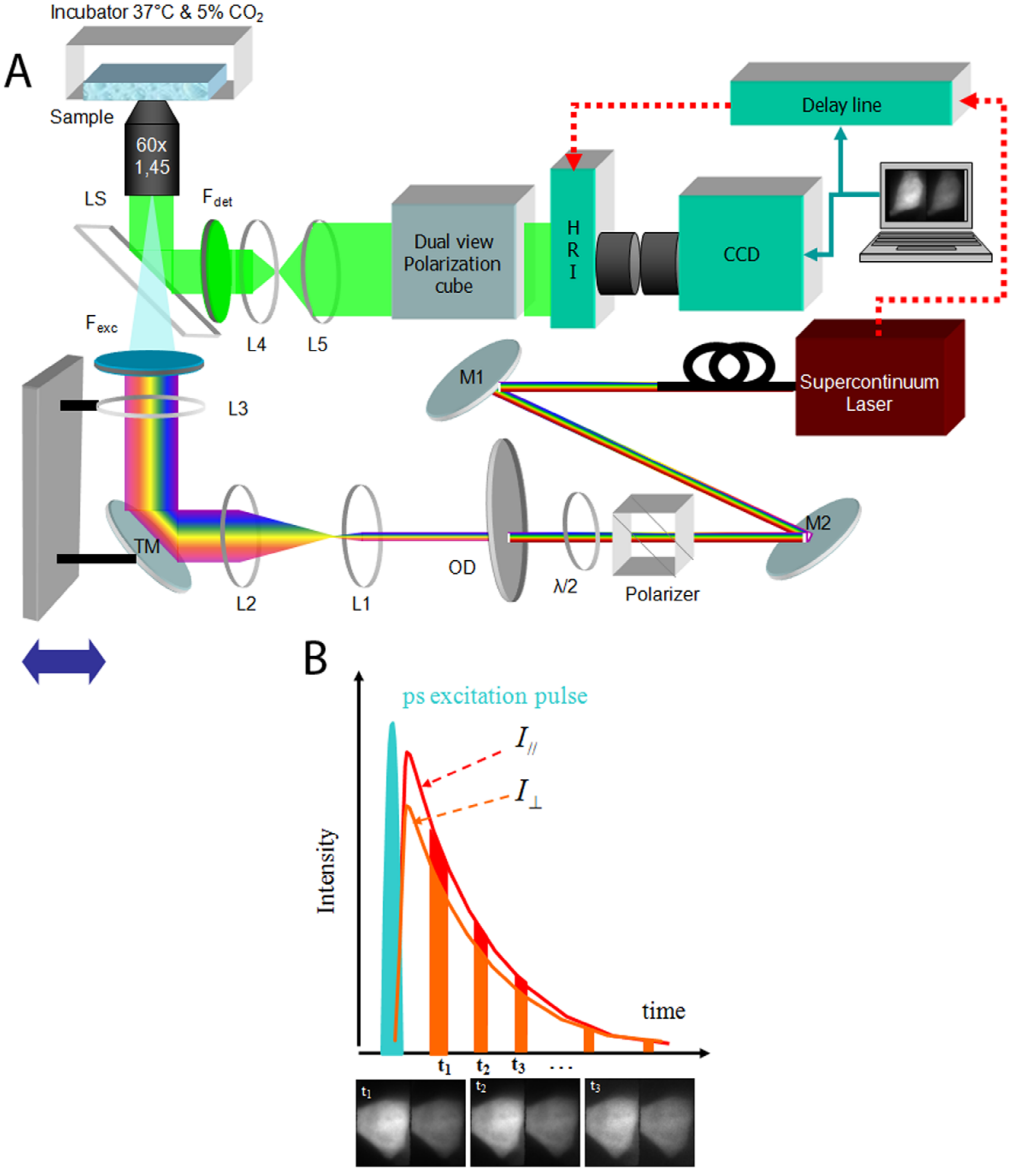
Schematic of the tr-FAIM coupled to a TIRFLIM set-up. *(A)* Schematic of the set-up. SC: supercontinuum fiber laser. M1 & M2: cold mirrors to filter the infrared part of the laser. OD: optical density to control the laser power, which is typically of 500 µW (measured in the back focal plane of the objective). L1 & L2: telescope to enlarge the beam to the field of view. F: filter. L3: lens to focus the beam in the back focal plane of the objective 60**×** (NA = 1.49). TM: mirror mounted on a translation stage to switch from an epifluorescence excitation to a TIRF excitation with different penetration depths. HRI: high rate imager. CCD: charge-coupled device camera. *(B)* tr-FAIM acquisition. Temporal gates are opened at different times after the laser pulse, resulting in a series of time-gated images for each polarization.

To prevent limitation in the choice of fluorophores, a picosecond supercontinuum fiber laser (SC400, Fianium Ltd, Southampton, UK) with a wide spectrum in the visible band (400–800 nm) was used. Since this laser is not linearly polarized, we used a Glan Taylor polarizer (Thorlabs, Newton, NJ) combined with a half waveplate (*λ/2*), to control the orientation of the linearly polarized output and to adjust it to the wavelength of interest (Fig. 1 *A*). Anisotropy experiments were performed in TIRF and epifluorescence excitation. To perform TIRF, a “through the objective configuration” was chosen, since we needed to have access to the sample [4]. Experiments were carried out with a TIRF Nikon objective 60**×** with a high NA of 1.49. For epifluorescence configuration, comparisons with low NA objectives were performed (DPLAN Olympus 10**×** NA = 0.3 and a DPLAN Olympus 40**×** NA = 1). The lateral position of the focused laser beam in the back focal plane of the TIRF objective was monitored with a motorized translation stage, thus enabling us to switch from epifluorescence excitation to a TIRF with different penetration depths from 0 nm to 300 nm (Fig. 1 *A*). An incubator (PeCon GmbH, Erbach, Germany) was implemented on the set-up so that all experiments were performed at 37°C and 5% CO_2_ (Fig. 1 *A*).

The fluorescence collected from the sample through the objective was split *via* a polarization-resolved imager (OptoSplit, Cairn Research Ltd., UK) with a polarization filter cube. Input fluorescence image was thereby divided into two images parallel and perpendicular to the excitation polarization, for the same region of interest. Image acquisition was performed in wide-field using a High Rate Imager (HRI, Kentech Ltd, Wallingford, UK) that was optically relayed to a CCD camera and synchronized to the laser pulse through a delay line. Temporal gates with different widths (from 200 ps to 2 ns with an increment down to 25 ps) could thus be opened at different times after the laser pulse to accurately sample the fluorescence decay for each polarization (Fig. 1 *B*). Starting from these time-gated series, fluorescence decays can be obtained for each polarization. For our experiments, we used a model with 30 time gates of 800 ps width to acquire the fluorescence decays for the intensities parallel 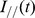 and perpendicular 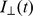 to the excitation polarization, each time gate corresponding to an average of 10 images (acquisition time for each time gate 2.5 s). For the fluorescence anisotropy decays, we adapted the number of time gates according to the remaining signal in the two channels compared to the background.

### Fluorescence Correlation Spectroscopy (FCS)

FCS was performed on a set-up previously described in [4]. For each fluorescein solution, fluorescence variations were assessed by collecting 10 sets of data for 30 s each. If photobleaching was detected during the 30 s collection, the sample was discarded. A mean auto-correlation curve was calculated for the non-photobleached samples. This mean FCS curve was fitted with OriginPro7 (OriginLab, Northampton, MA) software using an equation for 3D free diffusion of two populations:
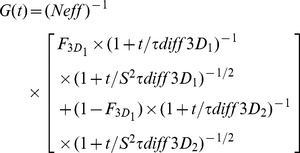
(1)With *t* representing time, *Neff* the average number of fluorescent molecules in the effective measurement volume, *τ_diff_* the diffusion time of a given population, *S* the structure parameter that characterized the shape of the detection volume and *F* the fraction of molecules of a given population. In this equation, one population (very fast diffusion times in the 10 µs range) corresponded to the non specific contribution of the afterpulse while the second population (much slower diffusion times, in the 60 to 1000 µs range) was related to the diffusion of fluorescein molecules in solution. FCS experiments gave us the diffusion time of fluorescein *τ_diff_*, which is linked to the environment viscosity 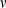
and to the hydrodynamic radius *r_h_* following the relations below [25].
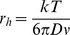
(2)With
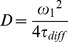
(3)and 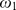 representing the full width at half maximum of the point spread function (PSF).

### Statistics

All statistical calculations were performed using GraphPadPrism 5.0 software (GraphPad, San Diego, CA).

### Theoretical Background: Fluorescence Anisotropy

When fluorophores are excited with polarized light, their fluorescence emission properties can yield information on the sample that cannot be extracted with intensity or lifetime methods. By evaluating the depolarized emission, fluorescence anisotropy can be probed [2].

Time-resolved anisotropy is defined by:
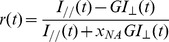
(4)Where 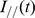 and 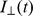 are the background subtracted fluorescence intensity decays with polarization parallel and perpendicular to the excitation polarization, respectively. The *G* factor accounts for the difference of transmission efficiencies of the optics for vertically and horizontally polarized light. *x_NA_* is an empirical correction factor that depends on the NA of the objective used. If objectives with numerical apertures below 0.9 are used, there is negligible depolarization induced by the objective which means that, considering a *G* factor equal to 1, the total fluorescence intensity is the sum of intensity of the polarization component parallel and of the two components perpendicular to the direction of the excitation polarization. In this case, *x_NA_* = 2. However, using objectives with large NA will induce a depolarization of the fluorescence collected through these objectives. Thus, these objectives will act as integrating sphere and collect fluorescence noise. This will lead to a redistribution of the intensities of fluorescence in the two polarization detection channels. *x_NA_* is thus not 2 anymore and has to be corrected to take into account the depolarization induced by the objective [26]–[29]. Experimental determination of *x_NA_* for our set-up was performed and compared to the theoretical approach [30].

The most common origin of depolarization is the rotational diffusion of molecules due to Brownian motion. It depends on the size and fluorescence lifetime of the molecules and also on their environment. Indeed, when the environment of a fluorophore is viscous, its rotational correlation time will increase with the viscosity [31]. Considering a spherical molecule that only undergoes rotation, the fluorescence anisotropy will follow a monoexponential decay, as given in Eq. 5:
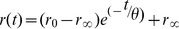
(5)
*r_0_* represents the initial anisotropy and only depends on the orientation of the fluorophore absorption and emission dipoles [31]. When they are parallel, *r_0_* will have a maximum value of 0.4. 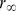 is the residual anisotropy which remains if the fluorophore rotation is hindered. *θ* is the rotational correlation time of the fluorophore and gives information about the viscosity of the cell environment according to the Stokes-Einstein equation:
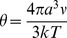
(6)Where *v* is the viscosity of the medium, *a* the hydrodynamic radius of the fluorophore considered to be spherical, *k* the Boltzmann constant and *T* the absolute temperature.

### Data Analysis

A program was developed under LabVIEW (National Instruments, Austin, TX) to acquire a stack of polarization-resolved images of the fluorescence decay. Images were first split according to the two polarizations using Image J plugin (Cairn Image Splitter). The variation of the anisotropy was then highlighted as 16-bits images by the LabVIEW program. Different parameters could be adjusted: the *G* factor, the background and the *x_NA_* factor. Due to the time-gated series obtained from the sampling of the fluorescence decay for each polarization, we can have the variation of the average fluorescence anisotropy over time using Eq. 4. OriginPro7 software was then used to fit these fluorescence anisotropy decays using exponential models.

Anisotropy maps represent the average anisotropy over all the time gates and were originally represented with a false colorscale to show interactions, whereas intensity images revealing structures were in black and white. To combine both information on the same image, we merged the two types of images in the HSV standard (Hue Saturation, Value), with intensity  =  hue and average anisotropy  =  value, to form a so-called photon-weighted anisotropy map where the average anisotropy is modulated by the intensity map.

## Results and Discussion

### Validation of the tr-FAIM Set-up

To validate our set-up, we used fluorescein solutions (6 µM) containing 0%, 28–35%, 44–50%, and 62–70% of glycerol, performed time-resolved fluorescence anisotropy measurements and deduced the rotational correlation time of fluorescein in these solutions. Each fluorescein solution was probed four times independently.

**Figure 2:**
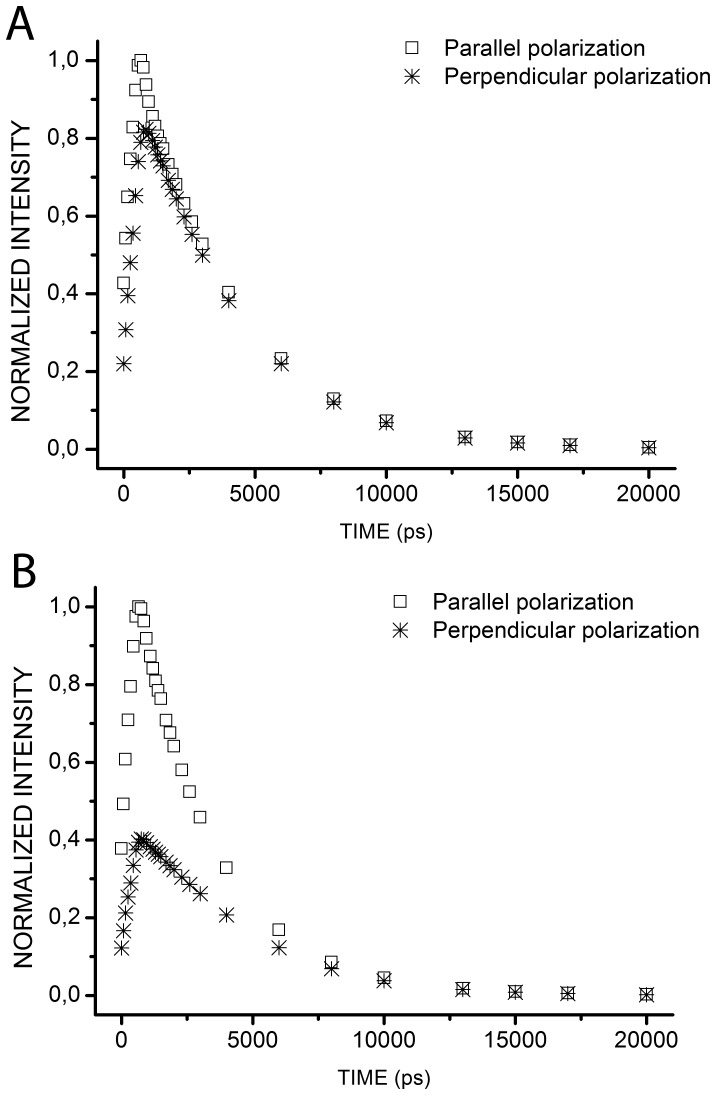
Fluorescence intensity decays obtained for parallel and perpendicular polarizations using objective 10× (NA = 0.3). Polarizations were normalized with the excitation polarization. Values obtained at each time gate for 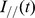 (*open squares*) and 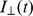 (*stars*) are represented. *(A)* non-viscous fluorescein solution. *(B)* fluorescein solution with a high viscosity (69–70% glycerol).

With the 0% glycerol solution, we determined *G^obj10^*, that reports the difference of transmission efficiency of the optics for the two polarizations, by calculating the ratio of 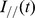 upon 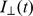. Fig. 2 *A* shows that for all solutions tails of the decays were overlapping. We confirmed that *G^obj10^* was not dependent on time from 400 ps to 8 ns after excitation and obtained an average value of 1.047±0.006 (n = 16 time gates). The standard deviation was sufficiently low (0.6%) to consider that *G^obj10^* was time independent, as it should be.

We extracted the fluorescence intensity decays for both polarizations from our images. Figure 2 shows examples of these decays obtained for a solution containing 0% glycerol (Fig. 2 *A*) and for a solution with high viscosity (69–70% glycerol) (Fig. 2 *B*) using objective 10**×** (NA = 0.3). For the non-viscous solution (0% glycerol), the curves obtained for 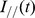 and 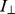
(t) overlapped rapidly as the fluorescein rotational correlation time is very short (*θ*∼200 ps) compared to its fluorescence lifetime (a few nanoseconds). Anisotropy resulting from Brownian motion was close to zero since no particular direction was favoured for the fluorescence emission. Figure 2 B shows the anisotropic aspect of fluorescence decays in viscous solutions.

**Figure 3:**
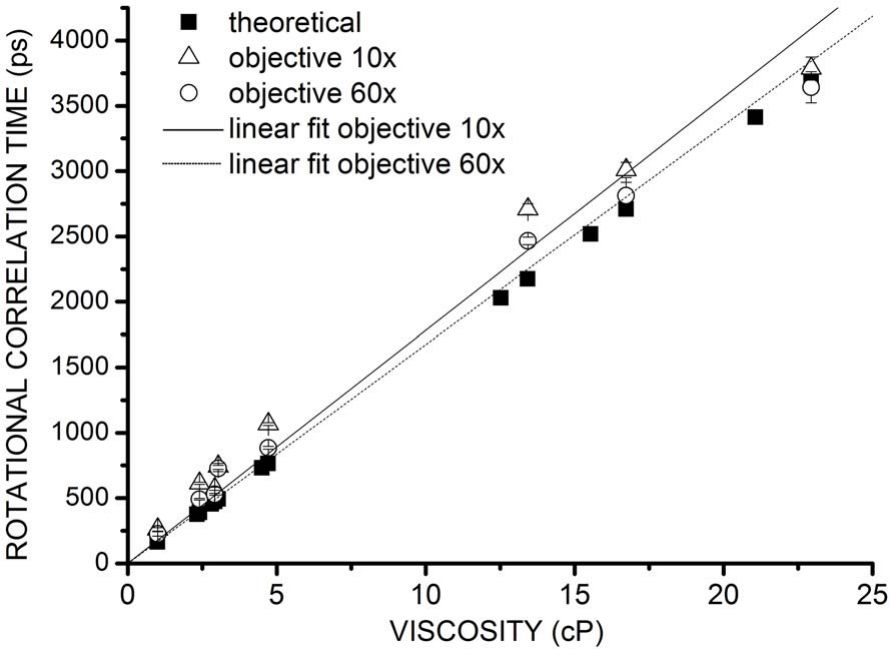
Variations of rotational correlation time of fluorescein as a function of viscosity. Theoretical rotation correlation times of fluorescein (*solid square*) and experimental ones are plotted for objective 10**×**, NA = 0.3 (*open triangle*) and for objective 60**×**, NA = 1.49 (*open round*).

Then, from these fluorescence intensity decays, we deduced the fluorescence anisotropy decay using Eq. 4. The anisotropy decay was fitted with a monoexponential model (Eq. 5) and the initial anisotropy, the rotational correlation time and the residual anisotropy were extracted. The rotational correlation time *θ* was then used to deduce the viscosity of the fluorescein environment from Eq. 6. To test the accuracy of the data obtained, the rotational correlation times calculated on our tr-FAIM set-up was plotted as a function of the viscosity measured with the refractometer (Fig. 3 and Table S1 in the Supporting Material). Since the NA of the objective 10**×** is 0.3, *x_NA_* equalled 2. Figure 3 shows rotational correlation time variations as a function of the viscosity of the solution for the experimental and theoretical data (calculated using Eq. 6, with the viscosities measured with the refractometer and assuming a fluorescein radius of 0.54 nm). Linear fit of the experimental data (R^2^ = 0.9937) gives a slope of 178±7 ps/cP. Using the Stokes Einstein equation (Eq. 6), the experimental fluorescein hydrodynamic radius was found to be 0.56 nm (with *k* = 1.38 × 10^−23^ m^2^·kg·s^−2^·K^−1^ and *T* = 295 K). This result is in agreement with the theoretical data and with previously described experimental measurements of fluorescein hydrodynamic radius [32]. Rotational correlation times of fluorophores can thus be accurately measured with our tr-FAIM microscope.

### Correction Factor for High Numerical Aperture Objectives

**Figure 4:**
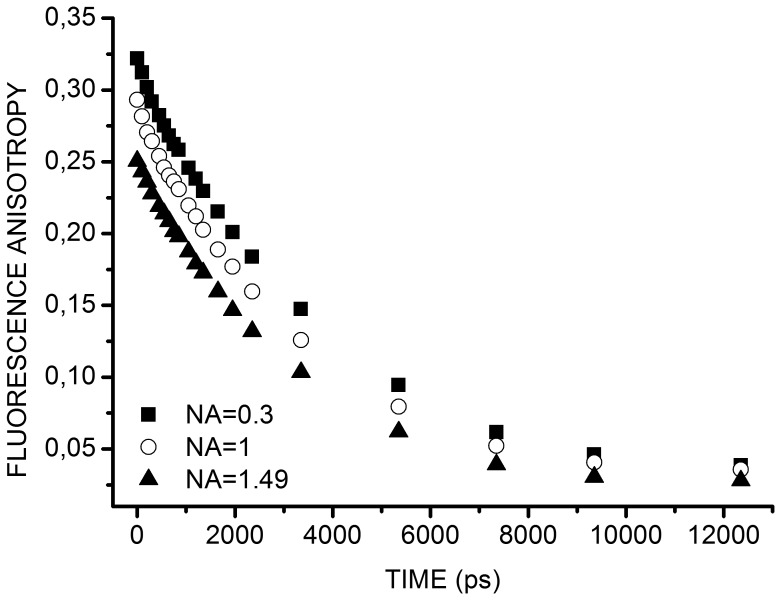
Fluorescence anisotropy decays of fluorescein acquired with objectives of different NA. Fluorescein solution of high viscosity (69–70% glycerol) was probed using objective 10**×**, NA = 0.3 (*solid square*), objective 40**×**, NA = 1 (*open round*) and objective 60**×**, NA = 1.49 (*solid triangle*).

Our aim was to measure the anisotropy at the plasma membrane of living cells using TIRF excitation requiring high NA objectives that induce a supplementary depolarization factor in the detection pathway [22]. We thus experimentally quantified *x_NA_* correcting factor associated with these objectives by calibrating the set-up on fluorescein solutions using objectives with different NA: the objective 10**×** NA = 0.3 mentioned above, an objective 40**×**, NA = 1 and the objective 60**×**, NA = 1.49 used for TIRF. Since fluorophores confined at the plasma membrane or in the cytoplasm of cells are in very viscous and heterogeneous environments [33], we thus studied highly viscous fluorescein solutions. Figure 4 shows variations of the fluorescence anisotropy decay, calculated using Eq. 4, with *x_NA_* = 2, obtained for a fluorescein solution containing 70% glycerol using the three objectives. Decrease of anisotropy was observed while increasing NA [29]. To correct this effect, we considered the measurements made with the objective 10**×** as our reference and corrected the fluorescence anisotropy decay obtained with the objective 60**×** accordingly. *x_NA_* can be deduced from the formula below obtained from re-arranging Eq.4 :
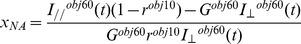
(7)Where 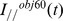 and 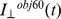 represent the fluorescence intensity for polarization parallel or perpendicular to the excitation respectively, measured with the objective 60**×** after subtracting the background. 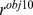
accounts for the fluorescence anisotropy measured with the objective 10**×** and 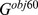 is the *G* factor measured with the objective 60**×**. This factor was calculated as for the objective 10**×** in non-viscous fluorescein solutions. Variations over time, from 400 ps to 8 ns after the excitation pulse, being minimal *G^obj60^(t)* = 1.042±0.004 (n = 16 time gates), *G^obj60^* was considered to be independent of time.

For each time gate, *x_NA_* was averaged on 10 acquisitions made on a fluorescein solution in a solution of 70% glycerol. *x_NA_(t)* was stable with an average value of 1.21±0.06, corresponding to a 61% correction compared to the standard value of 2 for low NA objective. These results confirmed that depolarization due to high NA of the objective deeply impact on time-resolved fluorescence anisotropy measurements and that correction is thus mandatory as has been observed before in single point measurements [28], [29], [34].

**Figure 5:**
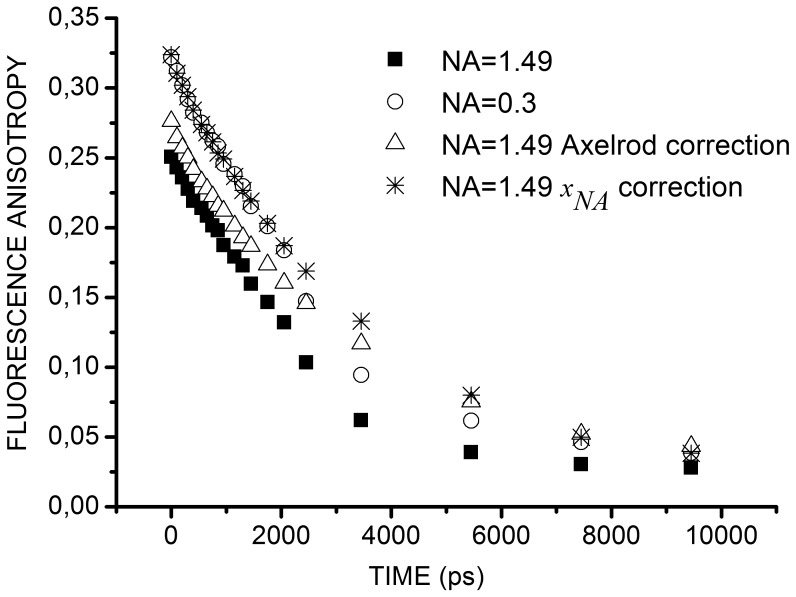
Comparison of different corrections of fluorescein fluorescence anisotropy decays. Fluorescence anisotropy decays of fluorescein in a highly viscous solution (69–70% glycerol) acquired with objective 10**×**, NA = 0.3 (*open round*) or objective 60**×** NA = 1.49 without correction (*solid square*), with Axelrod correction (*open triangle*), with *x_NA_* correction (*star*). The corrections offered by Axelrod were applied to our experimental data obtained with objective 60**×** and using *K_a_* = 0.31, *K_b_* = 0.03, *K_c_* = 0.66 in our case.

Our results are in agreement with the correction proposed by Axelrod in the case of high aperture observation of polarized emission [30]. Figure 5 shows that the fluorescence anisotropy of a highly viscous fluorescein solution measured with an objective 60**×** and corrected with *x_NA_*, is actually even closer to the fluorescence anisotropy measured with objective 10**×**, than the one measured with objective 60**×** and corrected according to Axelrod.

We measured the rotational correlation time of fluorescein in solutions of varying viscosities in epifluorescence excitation with objective 60**×** and with *x_NA_ = *1.2. Experimental and theoretical rotational correlation times were plotted as a function of the solution viscosity on Fig. 3. Data were collected from three independent experiments (Table S2). Linear regression slope of the experimental data was 167±5 ps/cP (R^2^ = 0.9966). We then deduced from Eq. 6 an experimental hydrodynamic radius of fluorescein of 0.54 nm, which is in agreement with the literature [32] and with the results obtained with the objective 10**×**.

In order to independently check the radius of fluorescein, measurements using Fluorescence Correlation Spectroscopy (FCS) were made on fluorescein solutions in the previous range of viscosities but with a hundred times lower concentration. The diffusion times obtained with FCS were plotted *versus* the solutions viscosity (Table S3). Linear regression (R^2^ = 0.9898) gave a slope of 0.0298 Pa^−1^ (*ω_1_* was 220 nm on our set-up). Equations 2 and 3 gave *r_h_* = 0.53 nm. Results obtained using FCS were consistent with the fluorescein radius of 0.54 nm measured with our time-resolved fluorescence anisotropy imaging microscope for both objectives 10**×** and 60**×** with *x_NA_* correction. Furthermore, these results are also in agreement with those found in the literature [32]. Convergence of these data indicated that using our correcting factor enabled us to obtain accurate anisotropy decays with high NA objectives.

### Impact of Correcting Factor on HomoFRET Measurements

Anisotropy measurements were performed on HEK293 cells transiently expressing cytosolic eGFP or a cytosolic eGFP-tandem (Fig. S1). eGFP is monomeric or dimeric whereas the eGFP-tandem is a constitutive dimer and was therefore used as a positive control for homoFRET. As both eGFP and eGFP-tandem are cytosolic, only epifluorescence measurements could be performed. We found *x_NA_* to be 1.2 (Fig. S2) highlighting the viscosity of the cytosol, in agreement with the literature [33].

**Figure 6:**
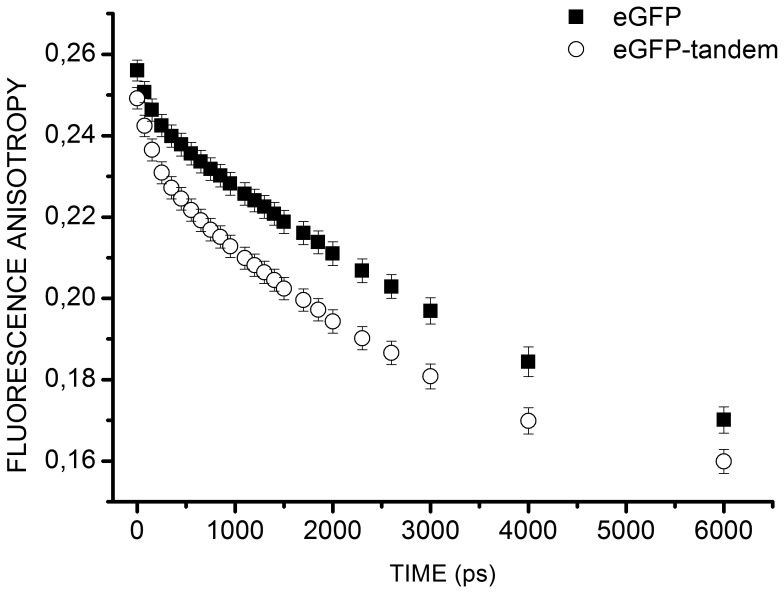
Mean fluorescence anisotropy variations over time for eGFP and eGFP-tandem. Mean fluorescence anisotropy decays for eGFP (*solid square*, n = 19 cells) and eGFP-tandem (*open round*, n = 21 cells) expressed in HEK293 cells.

eGFP is a large molecule with a rotational correlation time between 10–23 ns [7], [35], and a fluorescence lifetime around 2.4 ns [24], [31]. Figure 6 shows the mean fluorescence anisotropy decays obtained on cells expressing eGFP (19 cells) or eGFP-tandem (21 cells) from 2 independent experiments. The initial anisotropy was similar for the two proteins yet fluorescence anisotropy decreased more rapidly in the case of eGFP-tandem as compared to eGFP.

A decrease of eGFP fluorescence anisotropy can be linked either to rotation of eGFP in the excited state or to energy transfer, i.e. homoFRET. The data were thus fitted with a biexponential model [7]:
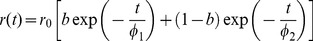
(8)where 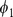 is a short depolarization time which accounts for the energy transfer and 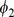 accounts for the rotational correlation time of the molecule. *b* represents the fraction of molecules in interaction. For cells expressing eGFP, the average depolarization time 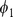 obtained was 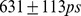 (n = 19). This value was used to adjust the fluorescence anisotropy decays obtained with eGFP-tandem, according to Eq. 8. We found an average proportion of 6.6±0.8% and 13.0±0.3% of eGFP and eGFP-tandem molecules in interaction respectively. These percentages of molecules in interaction were significantly different (unpaired t-test with Welch’s correction *p*-value<0.0001) revealing that while homoFRET occurred for eGFP, it was more frequent for eGFP-tandem. Average values obtained for 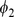 were not significantly different for eGFP and eGFP-tandem (19±1 ns and 20.6±0.5 ns respectively; unpaired t-test, *p*-value = 0.1699). These values are in agreement with the rotational correlation times measured for eGFP [7], [35].

Combined use of our tr-FAIM set-up and correcting factor enabled us to both evidence dimerization (or multimerization) of eGFP in the cytoplasm and differentiate dimerization patterns of eGFP and eGFP-tandem.

### Probing HomoFRET Differences of Wild-type and Constitutively Dimerizing APP-eGFP in the Cytoplasm

**Figure 7:**
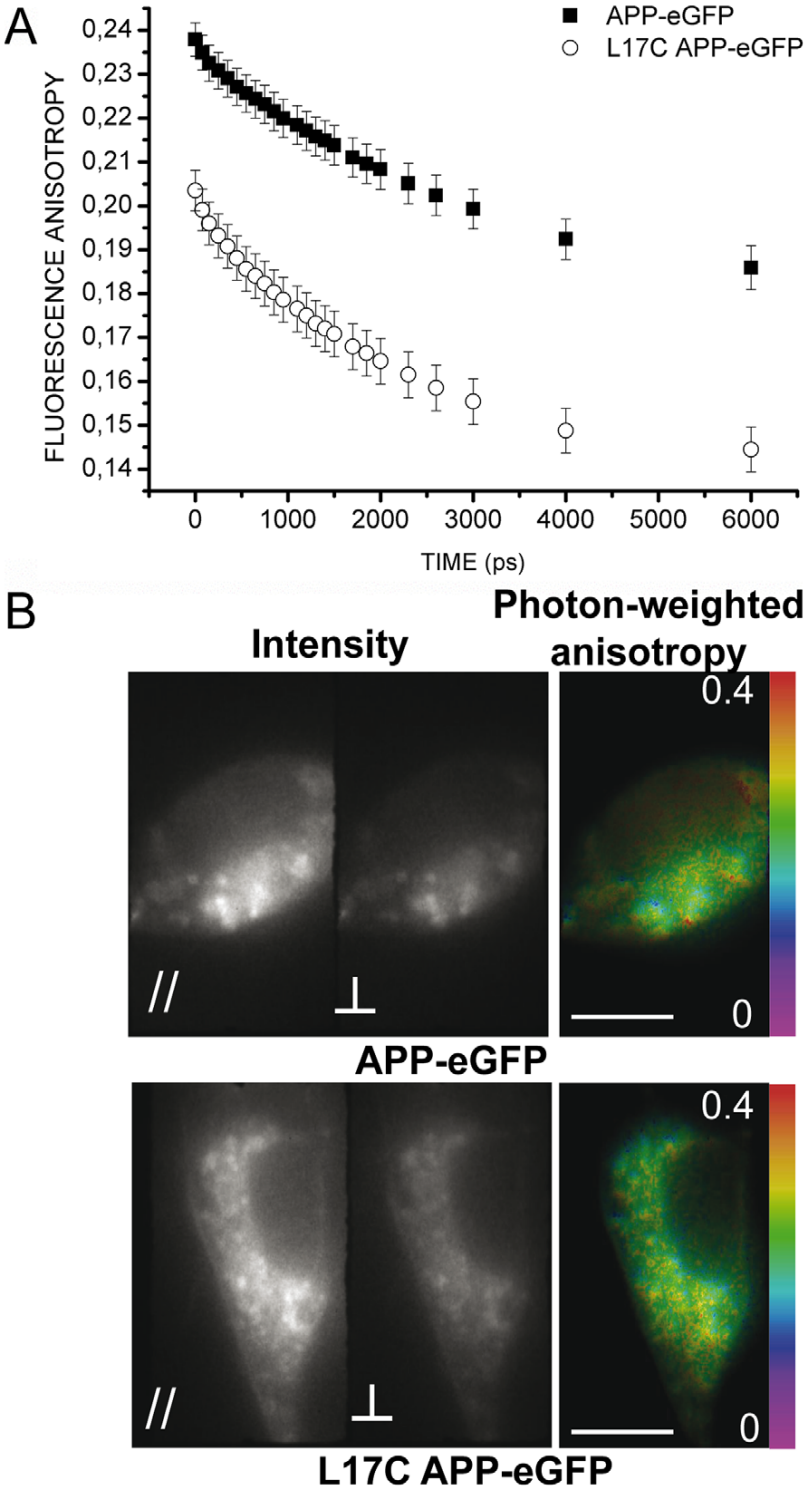
Anisotropy measurements for APP-eGFP and L17C APP-eGFP expressed in HEK293 cells in epifluorescence excitation. *(A)* Mean fluorescence anisotropy variation over time for APP-eGFP (*solid square*, n = 16 cells) and L17C APP-eGFP (*open round*, n = 27 cells). *(B)* Representative examples of mean fluorescence intensities with polarization parallel (*left panel*) or perpendicular *(central panel*) to the excitation one and mean photon-weighted fluorescence anisotropy map (*right panel*) for APP-eGFP (*upper panel*) or L17C APP-eGFP (*lower panel*). These images were averaged on all time gates. The scale bar represents 10 µm.

**Figure 8:**
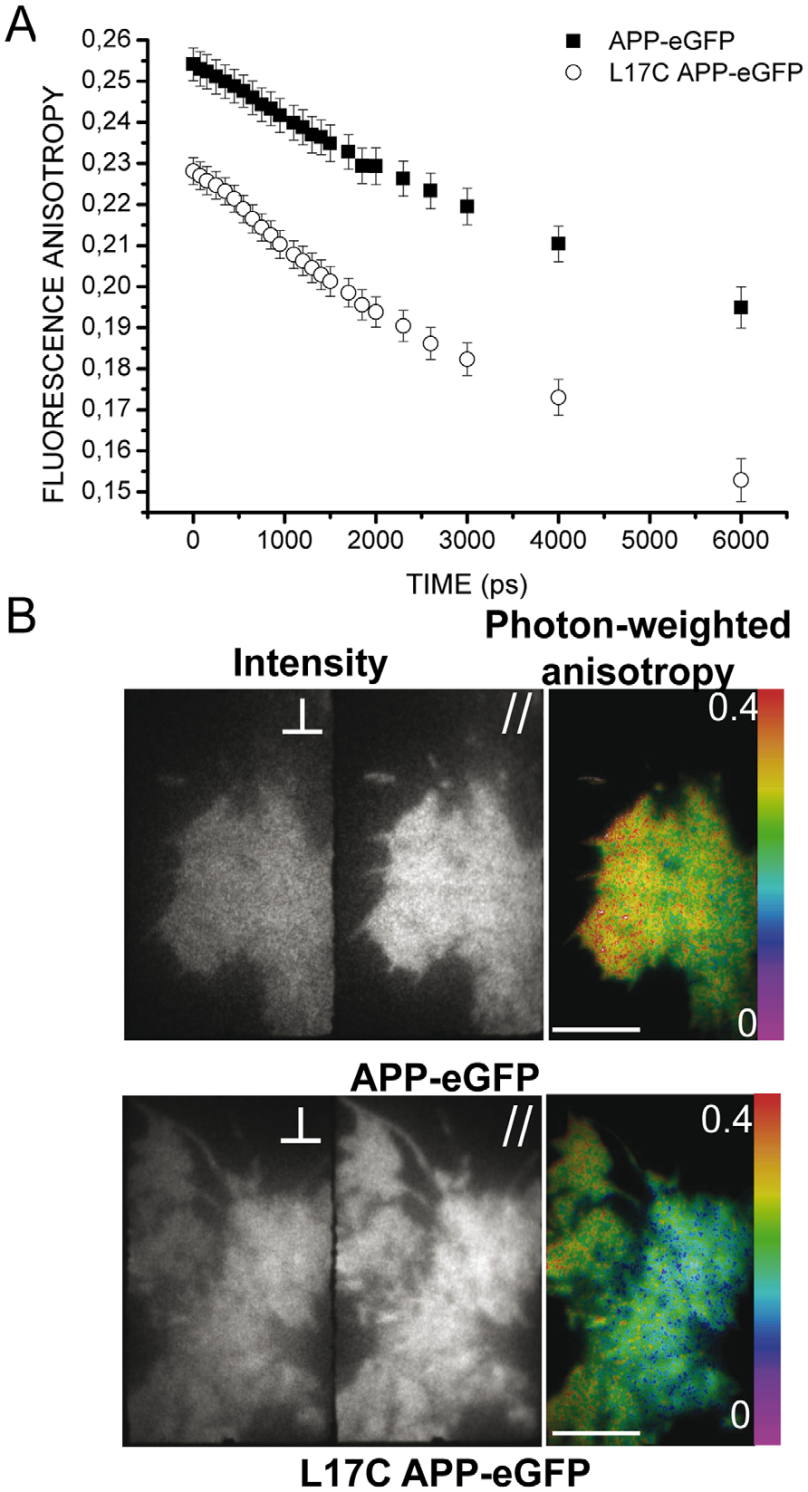
Anisotropy measurements for APP-eGFP and L17C APP-eGFP expressed in HEK293 cells in TIRF excitation. *(A)* Mean fluorescence anisotropy variation over time for APP-eGFP (*solid square*, n = 8 cells) and L17C APP-eGFP (*open round*, n = 9 cells). *(B)* Representative examples of mean fluorescence intensities with polarization parallel (*central panel*) or perpendicular *(left panel*) to the excitation one and mean photon-weighted fluorescence anisotropy map (*right panel*) for APP-eGFP (*upper panel*) or L17C APP-eGFP (*lower panel*). These images were averaged on all time gates. The scale bar represents 10 µm.

We then applied tr-FAIM to the homodimerization of APP, a key player in Alzheimer’s disease. We thus used a fluorescent APP construct, APP-eGFP, either in a wild-type (wt) or a mutated version (L17C). In L17C, the leucine at position 17 in the A*β* sequence was mutated to a cysteine. This mutation has been shown to induce the formation of covalent dimers as assessed by western blot (Fig. S3) and as previously described [19]. The two fluorescent APP-eGFP, wt and L17C, were similarly expressed both in intra-cellular vesicles (Fig. 7 *B*) and at the plasma membrane (Fig. 8 *B*). We thus tried to differentiate the dimerization patterns of wt APP-eGFP and L17C APP-eGFP on our set-up.

We performed time-resolved fluorescence anisotropy measurements on HEK293 cells expressing either wt APP-eGFP or L17C APP-eGFP in epifluorescence excitation. Figure 7 *A* shows the average fluorescence anisotropy decays obtained from 3 independent experiments. Figure 7 *B* shows the average intensities for polarizations parallel or perpendicular to the excitation and the photon weighted average anisotropy for a cell expressing APP-eGFP (*upper panel*) or L17C APP-eGFP (*lower panel*). The anisotropy measured at the first time gate was lower for L17C as compared to wt (Fig. 7 *A*). This could be due to a very fast energy transfer happening before the first measurement.

We first used a biexponential model (Eq. 8) to fit the fluorescence anisotropy decays obtained for each cell. We found short depolarization times for 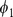 which accounted for the energy transfer and much longer correlation times for 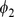 (>80 ns). These very long times could be due to hindered rotation of eGFP when linked to transmembrane APP and/or to preferred orientation of the dipoles when FRET was occurring. We thus gathered these parameters under a residual anisotropy 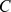 term and fitted our experimental data with:
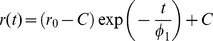
(9)


For cells expressing wt APP-eGFP or L17C APP-eGFP, we found an average value for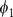 of 3.6±0.4 ns (n = 16 cells) and 2.4±0.1 ns (n = 27 cells) respectively. These depolarization times associated with energy transfer were significantly different (t-test with Welch’s correction, *p-*value = 0.0028), showing that we could quantitatively and statistically differentiate the energy transfer rates of dimerization of wt versus L17C APP-eGFP in epifluorescence. Faster energy transfer rate in the case of L17C APP-eGFP may be linked to structural changes induced by the L17C mutation.

### Probing HomoFRET Differences of Wild-type and Constitutively Dimerizing APP-eGFP at the Plasma Membrane

Time-resolved fluorescence anisotropy was then measured on HEK293 cells expressing wt APP-eGFP or L17C APP-eGFP in TIRF excitation. To achieve fluorescence anisotropy imaging in TIRF excitation, an s polarized excitation light was necessary to avoid the excitation of dipoles both parallel and perpendicular to the direction of the excitation polarization [36]. We thus turned the half waveplate by 45° to switch from a *p* to a *s* polarized excitation beam.

The *G* factor was assessed by calculating the 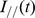/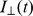 ratio for a fluorescein solution without glycerol. With epifluorescence excitation, we measured *G^obj60^_epi_ (t)* = 0.967±0.003 (averaged on 16 time gates, starting 400 ps after the excitation, before background noise). Measurements in TIRF excitation produced similar values of *G^obj60^_TIRF_ (t)* (0.965±0.002; two-tailed unpaired t-test, *p*-value = 0.053). Then we evaluated *x_NA_* with a *s*-polarized excitation light. The previous method used, based on correcting the anisotropy decay obtained with objective 60**×** with the one obtained with objective 10**×**, could not be applied to TIRF excitation since high NA objectives were required to perform TIRF excitation. Consequently, the determination of *x_NA_* was made in epifluorescent *s*-polarized excitation on a fluorescein solution of high viscosity (71.5% glycerol ie viscosity of 26 cP) with objectives 10**×** and 60×. By averaging the values on all the time gates, we obtained *x_NA_^polas^(t)* = 1.20±0.02. We thus found that, for objective 60×, *x_NA_* values for a *s*-polarized excitation light and for a *p*-polarized excitation light were similar.

Average fluorescence anisotropy decays for wt APP-eGFP or L17C APP-eGFP in TIRF excitation are presented in Fig. 8 *A*. Figure 8 *B* shows the average intensities for polarizations parallel or perpendicular to the excitation and the photon weighted average anisotropy for a HEK293 cell expressing APP-eGFP (*upper panel*) or L17C APP-eGFP (*lower panel*) in TIRF excitation. As for epifluorescence, fluorescence anisotropy measured at the first time gate was lower for L17C than for wt (Fig. 8 *A*). This may be caused by a very fast energy transfer between two L17C APP-eGFP at the plasma membrane. Fitting of the experimental TIRF anisotropy decays with equation 9 lead to average depolarization times 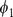
of 7.4±0.5 ns (n = 8 cells) and 5.4±0.3 ns (n = 9 cells), for cells expressing wt APP-eGFP or L17C APP-eGFP, respectively. 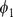 was significantly shorter for L17C than for wt APP-eGFP (t-test, *p*-value = 0.0025), highlighting that we could statistically differentiate homoFRET transfer rates, and thus homodimerization patterns, of wt *versus* L17C APP-eGFP at the plasma membrane.

### Conclusion

To study protein homodimerization at the plasma membrane of living cells, we implemented time-resolved Fluorescence Anisotropy Imaging (tr-FAIM) on a TIRFLIM microscope. We first validated our set-up by ensuring that we could accurately measure rotational correlation times of fluorophores. We then evaluated depolarization factors associated with transmission efficiency of the optics (*G*) and with high NA objective (*x_NA_*) both in solution and in the cytoplasm of cells for different fluorophores of varying size and lifetime excited with *s* or *p* polarized light. The experimentally determined *x_NA_* correction factor proved to be even more accurate than the theoretical one proposed by Axelrod [30] demonstrating that tr-FAIM measurements could be accurately performed using both low and high NA objectives on our set-up. This opens the way to measure rotational correlation times as well as homoFRET both in epifluorescence and in TIRF excitation.

Our tr-FAIM set-up enables us to probe homodimerization of proteins of interest with unrivaled accuracy and ease. (i) Acquisition can be performed on living cells. (ii) Acquisition is fast (same time range as for FLIM-FRET). (iii) We only need one type of fluorescent protein label, thus eliminating the artefacts of differential expression of the “classic” donor and acceptor-tagged proteins. This is also crucial when it comes to using primary cultured cells which are difficult to transfect. (iv) As there is only one tagged protein; it is easy to perform post-fixation multi-coloured immuno-cytochemistry analysis to go further in mechanism exploration. (v) Last but not least, we have access to the whole pool of homodimers, unlike classic FRET techniques.

We showed differences between the dimerization patterns of wild-type APP-eGFP and a construct bearing a L17C point mutation, forming constitutive dimers. Measurements were made on HEK293 cells expressing either APP-eGFP construct in epifluorescence and in TIRF excitation. APP-eGFP homoFRET was observed both at the plasma membrane and inside the cell. We also found that for both excitation conditions, energy transfer rates for mutant APP-eGFP were faster than for the wild-type, reflecting increased stability and structural changes in the disulfide linked dimer. We were thus able to statistically differentiate between the two types of homodimerization both at the plasma membrane and inside the cells. These differences might even be underestimated as an energy transfer faster than our time-resolution was suspected for mutant APP-eGFP.

Indeed, it was previously shown that APP dimerizes in mammalian cells both intracellularly [19], [37] and at the plasma membrane [38]. Here, using APP L17C mutant that displays higher dimerization than wild-type [19] we show that enhanced dimerization is found both inside the cells and at the plasma membrane. Our results are in good agreement with these previous studies.

Thus we were able to identify APP dimerization using anisotropy. However access to the proportions of dimers versus monomers still remains a challenge. Anisotropy has been used to study the oligomerisation of Ca^2+^/calmodulin-dependent protein kinase IIα (CaMKIIα), a cytosolic protein. In their study Thaler et al compared anisotropy decays of Venus tagged CaMKIIα with those of different oligomerization-controlled forms of fluorescent Venus [11].

Most studies addressing APP homodimerization focused on the interaction domains and the particular residues involved in dimerization [19], [37], [39], [40]. Mutations found in genetic forms of AD were found to destabilize APP homodimers [41].

The role of homodimerization in APP processing was extensively studied [18]–[21], , though controversial results were obtained depending on the methods and APP constructs used. Other studies relate a role for APP family members dimerization in trans-cellular adhesion [43], [44].

Using tr-FAIM, we were able to dynamically follow APP homodimerization in live eukaryotic cells both at the membrane and intracellularly in a non-invasive and non-destructive manner. One way to take advantage of these special features would be to address the effect of homodimerization on APP trafficking. We have previously shown that increasing plasma membrane cholesterol results in a relocalization of APP to cholesterol-rich membrane microdomains termed lipid rafts [4] followed by its increased endocytosis [4], [45]. It would be interesting to probe in neuronal primary cultures issued from control animals or AD models whether the micro-environment of lipid rafts is beneficial to the formation of APP homodimers and whether APP homodimers endocytosis is increased.

In conclusion, this original tr-FAIM set-up coupled to a TIRF microscope enables the dynamic follow-up of proteins homodimerization both at the plasma membrane and in the cytoplasm of living cells. Future applications include screening the effect of specific ligands on homodimers formation or on stages of proteins oligomerization in living cells.

## Supporting Information

Figure S1 **Mean fluorescence intensities and mean photon-weighted fluorescence anisotropy map for eGFP or eGFP-tandem.**
(DOC)

Figure S2 **Fluorescence anisotropy decays of eGFP acquired with objectives 10× (NA = 0.3) and 60× (NA = 1.49).**
(DOC)

Figure S3 **Effect of the L17C mutation on APP dimerization.**
(DOC)

Table S1 **Effect of viscosity on rotational correlation time of fluorescein measured with objective 10× (NA = 0.3).**
(DOC)

Table S2 **Effect of viscosity on rotational correlation time of fluorescein measured with objective 60× (NA = 1.49).**
(DOC)

Table S3 **FCS measurements of fluorescein diffusion time in different viscosities.**
(DOC)
