# Supplementary material for: Homodimerization of Amyloid Precursor Protein at the Plasma Membrane: A homoFRET Study by Time-Resolved Fluorescence Anisotropy Imaging
Source: PLoS One. 2012 Sep 4;7(9):e44434. doi: 10.1371/journal.pone.0044434 (PMC3433432; doi:10.1371/journal.pone.0044434)
Supplement: Figure S1 — Mean fluorescence intensities and mean photon-weighted fluorescence anisotropy map for eGFP or eGFP-tandem. (DOC) [file pone.0044434.s001.doc]

**SUPPORTING MATERIAL : Figure S1**

**
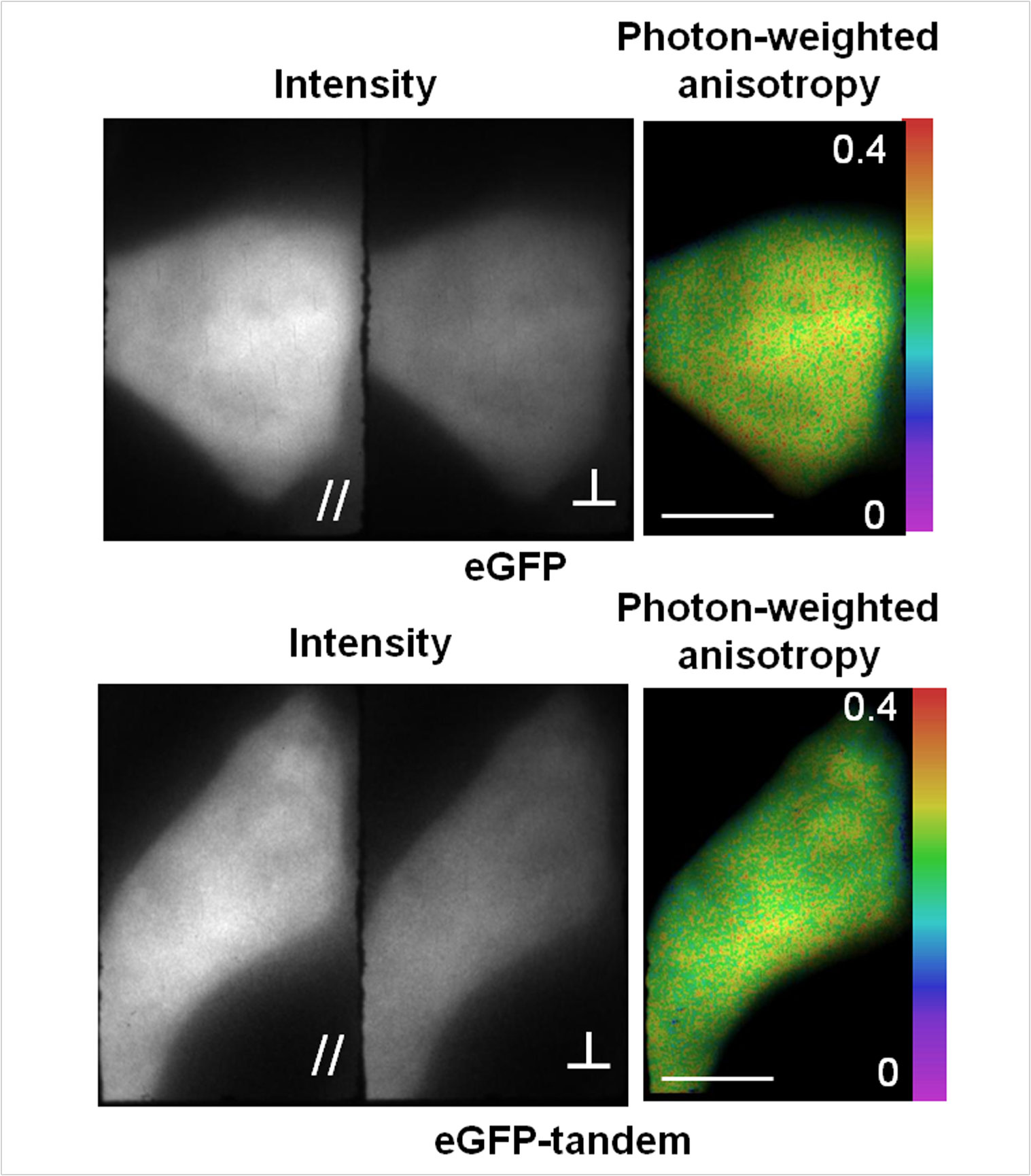
**

**Figure S1** : **Mean fluorescence intensities and mean photon-weighted fluorescence anisotropy map for eGFP or eGFP-tandem**.

Representative examples of mean fluorescence intensities with polarization parallel (*left panel*) or perpendicular *(central panel*) to the excitation one and mean photon-weighted fluorescence anisotropy map (*right panel*) for eGFP (*upper panel*) or eGFP-tandem (*lower panel*). These images were averaged on all time gates. **The scale bar represents 10 µm.**
