# Supplementary material for: Homodimerization of Amyloid Precursor Protein at the Plasma Membrane: A homoFRET Study by Time-Resolved Fluorescence Anisotropy Imaging
Source: PLoS One. 2012 Sep 4;7(9):e44434. doi: 10.1371/journal.pone.0044434 (PMC3433432; doi:10.1371/journal.pone.0044434)
Supplement: Figure S2 — Fluorescence anisotropy decays of eGFP acquired with objectives 10× (NA = 0.3) and 60× (NA = 1.49). (DOC) [file pone.0044434.s002.doc]

**SUPPORTING MATERIAL : Figure S2**

**
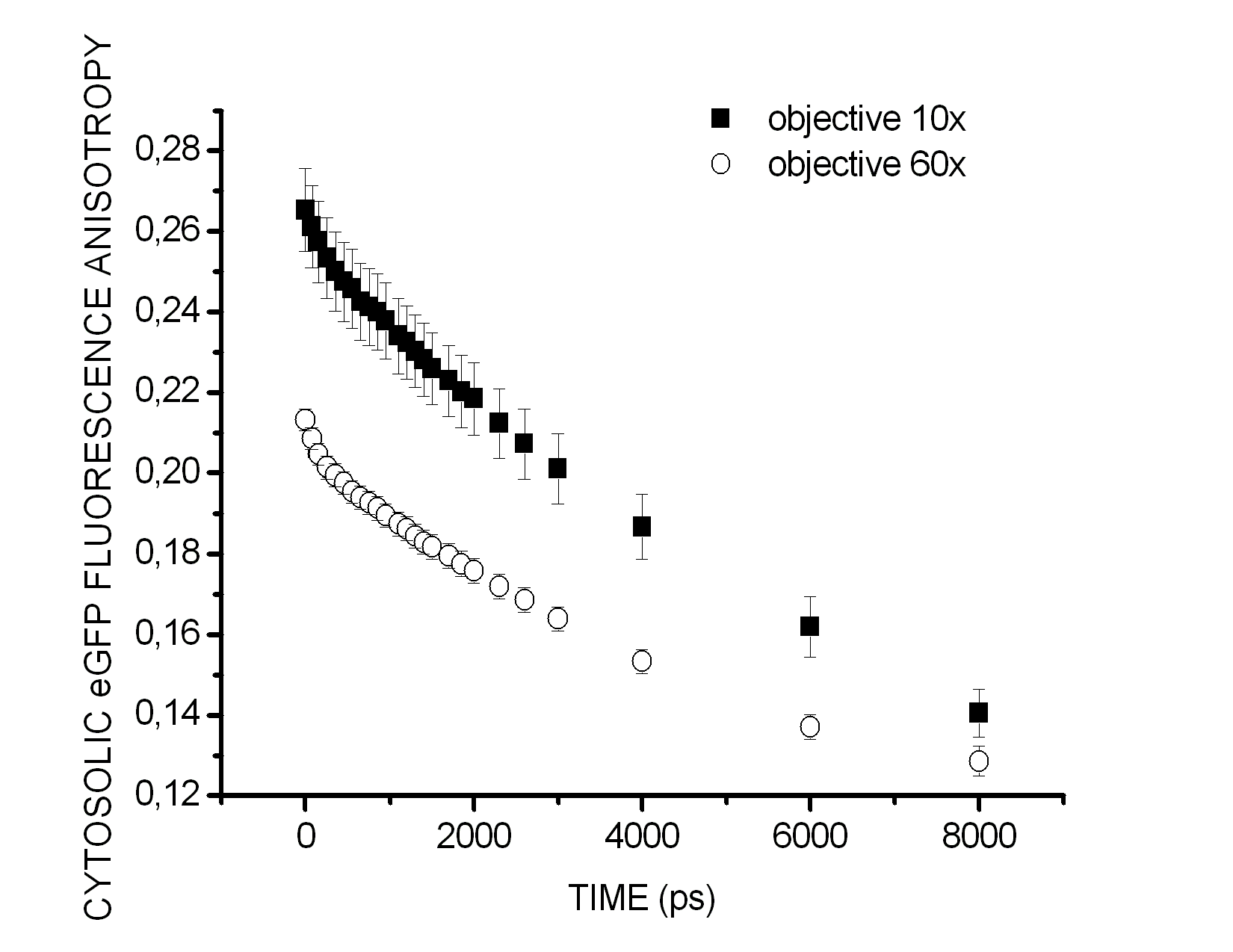
**

**Figure S2:** **Fluorescence anisotropy decays of eGFP acquired with objectives 10x (NA=0.3) and 60x (NA=1.49).**

Fluorescence anisotropy decays of eGFP expressed in HEK-293 cells calculated from measurements with objective 10x (average on about 60 cells) (*solid square*) and with objective 60x (average on 10 cells) (*open round*). For these results *x_NA_* was equal to 2.

As previously described for solutions, fluorescence anisotropy was found to decrease for higher NA objectives. *x_NA_* was consequently quantified by performing acquisitions on HEK-293 cells expressing cytosolic eGFP using either objective 10x or 60x. As for solutions, measurements made with the objective 10x were considered as our reference. We corrected individual fluorescence anisotropy decays obtained on a unique cell with the objective 60x, with the average fluorescence anisotropy decay obtained on cells (average on about 60 cells) with the objective 10x. A mean *x_NA_* value was deduced by averaging all the correcting factors previously determined for individual cells.
