# Supplementary material for: Homodimerization of Amyloid Precursor Protein at the Plasma Membrane: A homoFRET Study by Time-Resolved Fluorescence Anisotropy Imaging
Source: PLoS One. 2012 Sep 4;7(9):e44434. doi: 10.1371/journal.pone.0044434 (PMC3433432; doi:10.1371/journal.pone.0044434)
Supplement: Figure S3 — Effect of the L17C mutation on APP dimerization. (DOC) [file pone.0044434.s003.doc]

**SUPPORTING MATERIAL : Figure S3**

**
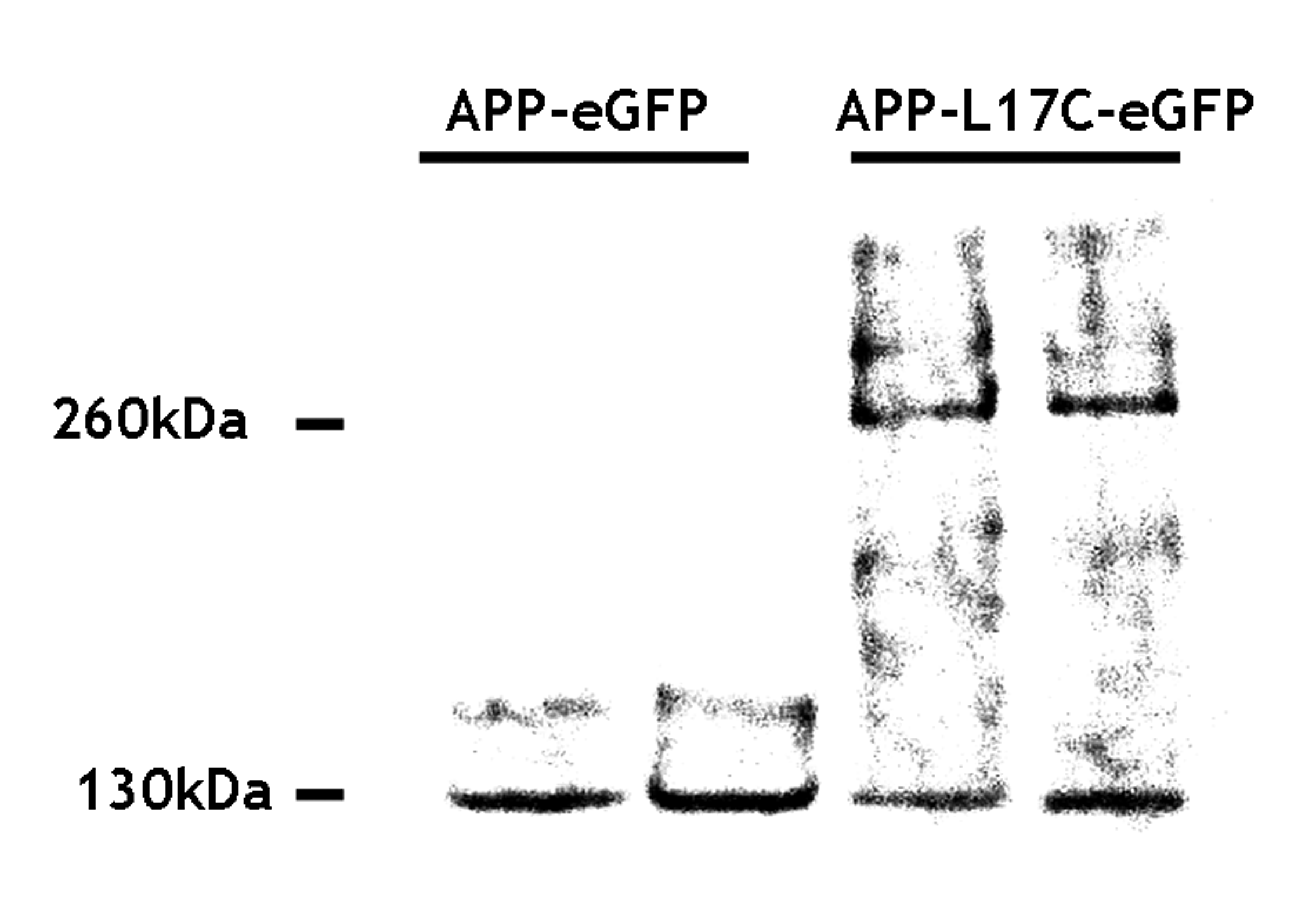
**

**Figure S3: Effect of the L17C mutation on APP dimerization.**

HEK-293 cells expressing either APP-eGFP (*left*) or L17C APP-eGFP (*right*) were analyzed by gel electrophoresis under reducing conditions and western blot with an anti-GFP antibody. In these conditions, only monomers (130 kDa) and covalent dimers (260 kDa) will be detected.

HEK-293 cells expressing either APP-eGFP or L17C APP-eGFP were washed twice, and proteins were extracted for 2 hours in a Tris buffer (0,05 M) containing 0,1 M NaCl, 5 mM EDTA, 1 % Nonidet P-40 and a protease inhibitor cocktail (Roche, Basel, Switzerland). Cells debris were removed by centrifugation at 13000g. Protein concentration was determined using Dc protein assay kit (BIO-RAD, Hercules, CA). For western blot analysis, 50 μg of total protein per lane were loaded on 7% Tris gels under reducing conditions. Nitrocellulose membranes (Whatman, Maidstone, UK) were probed with anti-GFP (Sigma-Aldrich, Saint Louis, MO). After incubation with horseradish-conjugated secondary antibodies (Molecular Probes, Carlsbad, CA), signal was revealed using Immobilon Western chemoluminescent HRP substrate (Millipore, Billerica, MA).

Monomers (migrating around 130 kDa) and dimers (migrating around 260 kDa) of APP show two bands corresponding to immature (lower molecular weight, N-glycosylated) and mature (N and O-glycosylated) species[1].

**1. Weidemann A, Konig G, Bunke D, Fischer P, Salbaum JM, et al. (1989) Identification, biogenesis, and localization of precursors of Alzheimer's disease A4 amyloid protein. Cell 57: 115-126.**
