# Supplementary material for: Homodimerization of Amyloid Precursor Protein at the Plasma Membrane: A homoFRET Study by Time-Resolved Fluorescence Anisotropy Imaging
Source: PLoS One. 2012 Sep 4;7(9):e44434. doi: 10.1371/journal.pone.0044434 (PMC3433432; doi:10.1371/journal.pone.0044434)
Supplement: Table S1 — Effect of viscosity on rotational correlation time of fluorescein measured with objective 10× (NA = 0.3). (DOC) [file pone.0044434.s004.doc]

**SUPPORTING MATERIAL : Table S1**

| Percentage of glycerol (%) | Viscosity of the fluorescein solution (cP) | Rotational correlation time (ps) (mean ± sd) |
| --- | --- | --- |
| 0 | 1.005 | 261±14 |
| 29 | 2.41 | 613±8 |
| 34 | 2.92 | 571±12 |
| 35 | 3.04 | 743±22 |
| 45 | 4.715 | 1067±10 |
| 63 | 13.43 | 2709±41 |
| 66 | 16.73 | 3009±58 |
| 70 | 22.94 | 3786±88 |

**TABLE S1. Effect of viscosity on rotational correlation time of fluorescein measured with objective 10x (NA=0.3)**
