# Supplementary material for: Homodimerization of Amyloid Precursor Protein at the Plasma Membrane: A homoFRET Study by Time-Resolved Fluorescence Anisotropy Imaging
Source: PLoS One. 2012 Sep 4;7(9):e44434. doi: 10.1371/journal.pone.0044434 (PMC3433432; doi:10.1371/journal.pone.0044434)
Supplement: Table S2 — Effect of viscosity on rotational correlation time of fluorescein measured with objective 60× (NA = 1.49). (DOC) [file pone.0044434.s005.doc]

**SUPPORTING MATERIAL : Table S2**

| Percentage of glycerol (%) | Viscosity of the fluorescein solution (cP) | Rotational correlation time (ps) (mean ± sd) |
| --- | --- | --- |
| 0 | 1,005 | 226±16 |
| 29 | 2,41 | 492±7 |
| 34 | 2,92 | 529±8 |
| 35 | 3,04 | 727±24 |
| 45 | 4,715 | 885±11 |
| 63 | 13,43 | 2466±29 |
| 66 | 16,73 | 2812±103 |
| 70 | 22,94 | 3642±119 |

**TABLE S2. Effect of viscosity on rotational correlation time of fluorescein measured with objective 60x (NA=1.49)**
