# Supplementary material for: Homodimerization of Amyloid Precursor Protein at the Plasma Membrane: A homoFRET Study by Time-Resolved Fluorescence Anisotropy Imaging
Source: PLoS One. 2012 Sep 4;7(9):e44434. doi: 10.1371/journal.pone.0044434 (PMC3433432; doi:10.1371/journal.pone.0044434)
Supplement: Table S3 — FCS measurements of fluorescein diffusion time in different viscosities. (DOC) [file pone.0044434.s006.doc]

**SUPPORTING MATERIAL : Table S3**

| Range of percentage of glycerol (%) | Viscosity range of the fluorescein solution (cP) | Diffusion time (ms) |
| --- | --- | --- |
| 0 | | 1.05 | | --- | | 0.06 |
| 25-26 | 2.095-2.167 | | 0.18 | | --- | |
| 51-52 | 6.396-6.764 | | 0.3 | | --- | |
| 74-75 | 28.96-31.62 | | 0.94 | | --- | |

**TABLE S3. FCS measurements of fluorescein diffusion time in different viscosities**
